# Evoked slow oscillations and dynamic network reorganization after stroke

**DOI:** 10.1093/braincomms/fcaf391

**Published:** 2025-10-16

**Authors:** Caroline Tscherpel, Maike Mustin, Nils Rosjat, Lea-Theresa Mais, Ulf Ziemann, Gereon R Fink, Silvia Daun, Christian Grefkes

**Affiliations:** Goethe University Frankfurt, Department of Neurology, University Hospital Frankfurt, Goethe University, 60596 Frankfurt am Main, Germany; Medical Faculty, University of Cologne, and Department of Neurology, University Hospital Cologne, 50937 Cologne, Germany; Cognitive Neuroscience, Institute of Neuroscience and Medicine (INM-3), Research Centre Jülich, 52248 Jülich, Germany; Goethe University Frankfurt, Department of Neurology, University Hospital Frankfurt, Goethe University, 60596 Frankfurt am Main, Germany; Cognitive Neuroscience, Institute of Neuroscience and Medicine (INM-3), Research Centre Jülich, 52248 Jülich, Germany; Goethe University Frankfurt, Department of Neurology, University Hospital Frankfurt, Goethe University, 60596 Frankfurt am Main, Germany; Department of Neurology & Stroke and Hertie Institute for Clinical Brain Research, Eberhard-Karls-University Tübingen, 72076 Tübingen, Germany; Medical Faculty, University of Cologne, and Department of Neurology, University Hospital Cologne, 50937 Cologne, Germany; Cognitive Neuroscience, Institute of Neuroscience and Medicine (INM-3), Research Centre Jülich, 52248 Jülich, Germany; Cognitive Neuroscience, Institute of Neuroscience and Medicine (INM-3), Research Centre Jülich, 52248 Jülich, Germany; Goethe University Frankfurt, Department of Neurology, University Hospital Frankfurt, Goethe University, 60596 Frankfurt am Main, Germany

**Keywords:** diaschisis, neuroplasticity, small-worldness, modularity, randomness

## Abstract

A focal ischemic lesion is thought to alter neuronal activity beyond the area of structural damage, thereby interfering with the whole network architecture. Here, we used a combination of transcranial magnetic stimulation and electroencephalography in conjunction with dynamic connectivity analyses and graph theory to study alterations and reorganization of cortical connectivity in a cohort of 41 patients longitudinally after stroke. We found a link between an increase in low-frequency coupling in the delta band and alterations in neural information processing in the first weeks after stroke and their relevance for motor outcome >3 months later. We demonstrated that stroke enhances slow activity and delta coupling between frontocentral and parietal regions. In addition, we observed a loss of the physiological network architecture with a decrease in small-worldness and modularity in the delta frequency, implying that a focal ischemic lesion interferes with both cortical information integration and functional segregation within the first weeks after stroke. While we found a link between bifrontal coupling in the alpha spectrum and the degree of the motor deficit in the early post-acute phase, the amount of small-worldness disruption early after stroke indicated the motor outcome in the follow-up session. In contrast, recovery of motor function and cortical reorganization after >3 months post-stroke were paralleled by the normalization of increased low-frequency coupling and a reinstatement of the complex network structure featuring a modular and small-world topology.

## Teaser

Tscherpel *et al*. showed that increased frontoparietal low-frequency coupling and disrupted network architecture early post-stroke reflect diaschisis and thalamocortical dysfunction. Functional recovery was paralleled by normalized delta coupling and restored network topology. By linking oscillatory properties to alterations in connectivity post-stroke, this study adds to the understanding of remote lesion effects.

## Graphical Abstract

**Graphical Abstract:**
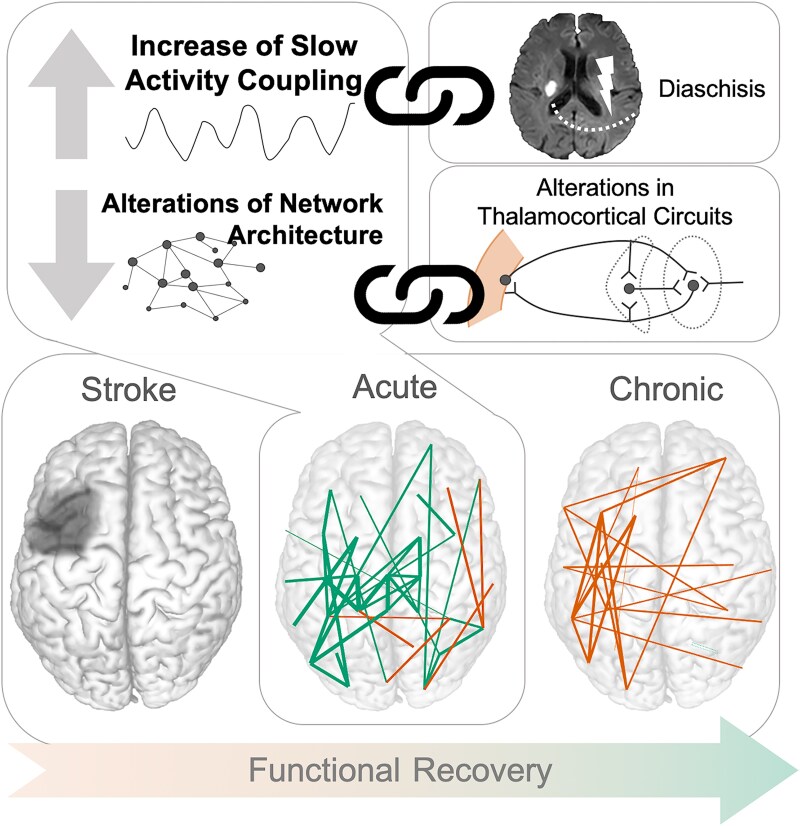

## Introduction

Graph theory has equipped network neuroscience with a language to describe and interpret the brain’s complex network organization.^1,2^ For structural and functional brain networks, in human and animal models, functional segregation and integration are two contrary yet complementary fundamental organizational principles of the human brain.^3^ A segregated neuronal architecture with distinct, specialized and highly intraconnected subdomains has been conclusively shown to be evident at multiple scales in neural networks.^4,5^ However, the synchronized processing between the local activity of various functional domains, i.e. functional integration, is equally crucial for effective network function. Hence, the balanced interplay between segregation and integration forms the basis of brain functionality and cognitive abilities.^3,6^

Focal stroke lesions affect the coordinated and balanced pattern of densely intraconnected and sparsely interconnected local modules of nodes, i.e. small-world topology.^7-10^ There is ample evidence of substantial alterations in network topology in both the ipsi- and contralesional hemispheres’ connectome post-stroke as derived by structural^11^ and functional magnetic resonance imaging (fMRI),^12,13^ forming the understanding of stroke as a large-scale network disorder with a focal stroke-induced lesion leading to widespread disruptions of interconnected areas distant from the lesion site.^14,15^ Correspondingly, the concept of diaschisis was coined over a century ago and revived in the context of connectional diaschisis by advancements in the understanding of complex network dynamics.^14,16^

However, studies investigating brain reorganization at the global network level reported conflicting results. While some studies suggested that stroke disrupts the balance between local processing and global functioning,^12,17,18^ resulting in a stroke-associated network dysfunction, other findings indicated an increase in global network communication to facilitate information transfer.^19^ It, thus, further remains elusive whether alterations in brain networks are a consequence of lesion-induced disconnection and hence may occur in the context of diaschisis, or result from plasticity mechanisms within the framework of vicariation supporting the recovery of function.

In this context, it needs to be considered that the fMRI BOLD signal provides only an indirect measure of neuronal activity, reflecting slow-evolving hemodynamic responses not optimally suited to portray the temporal dynamics of neural oscillations typically occurring in the range of milliseconds.^20^ By contrast, EEG directly captures the electrical signal generated by neuronal activity. Furthermore, it also comprehensively maps the entire spectrum of neuronal oscillatory activity, which has frequently been shown to be altered in stroke.^21-24^ Notably, the classical notion of electrophysiological recordings post-stroke is an increase in slow activity in association with a decrease in faster oscillations.^21,25,26^

Studies of resting-state activity, assessed with either fMRI or EEG, are particularly useful in stroke patients with motor deficits, as interindividual differences in performance or impairment do not influence the acquired signals. However, these spontaneous fluctuations of neural activity and connectivity only allow an indirect link to function.^27^

Here, applying a highly specific temporally and spatially precise neural perturbation may allow for the systematic and causal exploration of the resulting spatiotemporal dynamics.^28,29^ Combining transcranial magnetic stimulation (TMS) with EEG is a non-invasive perturb-and-measure approach in humans to quantify whole-brain cortico-cortical connectivity by probing activity propagation within interconnected networks, inducing synchronization of distant cortical areas and changes in functional dynamics at an individual and millisecond level.^29-33^ It has been shown that TMS–EEG can derive signals irrespective of their presence in resting-state EEG.^34^ Particularly relevant in this context, TMS–EEG signal changes follow spatial network dynamics measured by fMRI, indicating a common neural substrate.^20^

Recent work involving TMS–EEG has provided the fundament for the notion that slow waves are related to impaired signal complexity and thus neuronal network interactions.^22,24,35^ However, a comprehensive characterization of stroke-induced network alterations comprising the topological structure as well as the organizational principles still needs to be elucidated to link network pathology after stroke to stroke-associated EEG slowing, which we inferred to be interconnected. We hypothesized that by using TMS–EEG-derived connectivity, it would be possible to uniquely draw a link between graph-theoretic network analyses on dynamic networks and the functional layer of oscillatory properties, allowing new insights into the behavioral relevance of stroke-induced network alterations.

To this end, we used TMS–EEG to probe network changes in a cohort of 41 patients recovering from unilateral motor deficits due to a first-ever stroke in comparison to a group of healthy participants. To relate network topology to stroke reorganization and recovery of functional deficits, we analyzed longitudinal TMS–EEG and behavioral data from the first week after stroke until the early chronic stage after >3 months post-stroke.

## Materials and methods

### Participants

Forty-one stroke patients (37 right-handed, 6 females) were recruited and examined at the Department of Neurology, University Hospital of Cologne. Patients were included based on the following inclusion criteria: (i) first-ever ischemic stroke with (ii) ≤14 days elapsed from onset and resulting in (iii) unilateral upper limb deficit. Exclusion criteria were (i) contraindication to TMS,^36^ (ii) bihemispheric infarcts and (iii) hemorrhagic stroke (please see Supplementary Table 1 for further patient characteristics and Supplementary Fig. 1 for lesion overlap). In the early chronic phase, i.e. after >3 months, 35 patients underwent a behavioral follow-up visit, and 28 patients also participated in a second TMS–EEG assessment [mean: 138.3 ± 26.6 standard deviation (SD); range: 99–183 days]. Fifteen age-matched healthy participants without any history of neurological or psychiatric disease (14 right-handed, 2 females) served as a control group (age: patients: 65.1 ± 10.9, 49–87 years; healthy participants: 65.4 ± 5.8, 51–76 years; *P* = 0.93). Of note, data were previously included in Tscherpel *et al*.^22,37^ The current work builds on the findings of slow activity after stroke presented in the former publications; however, neither of them addresses the TMS–EEG-derived connectome and its organizational features post-stroke. Thus, there is no overlap in the analyses, as we did not incorporate any connectivity analyses before, and all analyses presented here are completely novel. All participants had provided informed written consent before inclusion in the study. The ethics committee of the Medical Faculty of the University of Cologne approved the study (file no. 17–244), carried out following the Declaration of Helsinki (October 2013).

The following motor and clinical scores were assessed within (i) the first 2 weeks and (ii) >3 months post-stroke: stroke severity and the global neurological impairment were assessed by the NIHSS (https://www.ninds.nih.gov/sites/default/files/documents/NIH-Stroke-Scale_updatedFeb2024_508.pdf); the Action Research Arm Test (ARAT) was used to evaluate gross and fine upper limb functions including pinch, grasp and grip; the Motricity Index (MI) classified muscular strength in the proximal, middle and distal joints of arms and legs; and maximum grip strength was determined by a vigorimeter (KLS Martin Group, Germany).

### Experimental procedure and data collection

A focal 70 mm figure-of-eight alpha coil connected to a Magstim Super Rapid^2^ stimulator (The Magstim Co. Ltd, Whitland, UK) delivering a biphasic current waveform was used for neuronavigated TMS.

To test the integrity and functionality of the motor network after stroke, we used the ipsilesional primary motor cortex (M1) as the stimulation target defined by the electrophysiological motor hotspot (Supplementary Material I). The resting motor threshold (RMT), i.e. the minimum stimulator intensity needed to evoke a motor evoked potential (MEP) with an amplitude of 50 µV in 5 out of 10 trials, was individually defined using the TMS Motor Threshold Assessment Tool 2.0 (http://www.clinicalresearcher.org/software.html). In 23 patients (MEP-negative patients), we could not determine an MEP in response to the stimulation of the ipsilesional M1, even with maximal stimulator output, due to the lesion-induced disruption of the corticospinal tract. Here, the RMT of the contralesional M1 served as a reference for the individual threshold.^22^ Additionally, in these cases, the ipsilesional M1 was anatomically identified as the hand knob on the precentral gyrus using the neuronavigation system.^38^ Notably, due to this procedure, the stimulation intensity did not differ, neither between patients (60.4 ± 12.3% maximum stimulator output (MSO) and healthy subjects (62.0 ± 14.8% MSO) (*P* = 0.07) nor between MEP-negative (59.8 ± 12.6% MSO) and MEP-positive patients (61.3 ± 12.0% MSO) (*P* = 0.7). During the second measurement, we re-assessed the individual RMT, yet stimulation intensities did not differ significantly between the first (61.0 ± 13.4% MSO) and the second session (58.3 ± 9.6% MSO) (*P* = 0.3).

TMS–EEG was recorded using a TMS-compatible, 64-channel EEG system. The EEG signals were sampled at 5 kHz with a resolution of 0.1 µV, high-pass filtered at 0.1 Hz and low-pass filtered at 1 kHz. The impedance of all electrodes was kept constantly below 5 kΩ. For each TMS–EEG session, we collected at least 100 trials of single TMS pulses with an interstimulus interval randomly jittered between 6.5 and 8.0 s and a stimulation intensity of 80% RMT (Supplementary Material II).

### Data analysis

#### Preprocessing

EEG responses to TMS were visually inspected, and channels and trials with bad signal quality or contaminated by muscular or ocular artefacts, as well as segments containing discharges, were manually rejected.^22,34,39^ Recordings with either <90 artefact-free trials or >10 poor channels were excluded from further analyses. Data processing was carried out as described in Tscherpel *et al*.,^22^ including band-pass, band-stop filtering and down-sampling (see Supplementary Material III). For all analyses, data from patients with right-hemispheric lesions (*n* = 23) were flipped along the midsagittal plane so that the lesioned side and, thus, the stimulation site corresponded to the left hemisphere in all participants.^40^ Correspondingly, data from healthy participants, in which the right hemisphere was tested, were also flipped to account for systematic effects of hemispheric differences.

#### Construction of phase-locking networks

Before connectivity analysis, EEG data were spatially filtered using the surface Laplacian (http://psychophysiology.cpmc.columbia.edu/Software/CSDtoolbox), which is considered to substantially reduce the effect of volume conduction by enhancing localized activity and suppressing diffused signals and, thus, allowing the analysis of electrodes close to the region of interest.^41,42^ Of note, edge electrodes were excluded from the analyses, leading to 39 electrodes for further investigation.

Subsequently, data were decomposed into the time–frequency domain using the Morlet wavelet transform with five cycles as a balanced solution between temporal and spectral resolution, enabling dynamic frequency analysis.^43^ Amplitude and phase information were further analyzed using customized Matlab scripts based on the open-source Dynamic Synchronization Toolbox (https://openresearchsoftware.metajnl.com/articles/10.5334/jors.394). We quantified the connection between two brain regions by the synchronization between the activity of two sites as determined by the single-frequency phase-locking value (PLV) at the sensor level; adapting the PLV defined in Lachaux *et al*.,^41,42^ considering the four frequency bands: δ (1–4 Hz), θ (4–8 Hz), α (8–12 Hz) and β (13–30 Hz) separately. Based on Rosjat *et al*.,^41^ the significance of PLVs was set by comparing the mean PLV obtained for each pair of electrodes at each time point and frequency band with its pre-stimulus baseline value, generated by normally distributed random values with the same mean and SD as the EEG signal at any time point during the baseline interval. Correspondingly, we used a pointwise *t*-test with a significance level of *P* < 0.05, FDR-corrected for multiple comparisons. To test whether changes averaged over frequency bands relative to pre-stimulus baseline differ significantly either between stroke patients and healthy controls or between the acute and chronic phase, we compared PLV values obtained for each pair of electrodes at each time point of the post-stimulus interval of interest, i.e. 0–600 ms, between groups. To this end, we used *t*-tests with a significance level of *P* < 0.05, FDR-corrected for multiple comparisons. The corrections were performed concerning the number of time points, groups and electrodes (see Supplementary Material IV).^41^

#### Applying graph theoretical measures

After the construction of the PLV-networks for each frequency band, undirected graphs with nodes formed by the 39 sensors and edges representing PLV values were constructed and analyzed using graph theoretical metrics as implemented in the Brain Connectivity Toolbox (https://sites.google.com/site/bctnet/).^44^ Based on previous publications applying graph theory on networks after stroke, we used a threshold of 0.5 to convert connectivity matrices (significant PLV matrices) into weighted networks, which means that PLV values higher than the applied threshold were referred to as edge weights.^12^ Depending on the network measure (see below), we have either utilized binary network matrices or weighted networks, focusing on differentiated and complementary aspects of the network organization.^44^

Notably, we investigated dynamic networks that are defined by a set of graphs and the time points at which they occur to account for time-varying connectivity patterns.^45^ Besides, we also analyzed static networks, which constitute aggregated graphs that consider the number of occurrences of each edge.

We confined our analyses predominantly to parameters characterizing the whole network. However, based on the finding of increased ipsilesional frontoparietal connectivity after stroke, we next analyzed connections of the ipsi- and contralesional networks separately using only sensors on the left or right hemisphere and edges within the centroparietal (FC1-FC3-C1-C3-CP3-P1-P3/FC2-FC4-C2-C4-CP4-P2-P4) or frontal network (Fz-F1-F3-AF3/Fz-F2-F4-AF4). In detail, we assessed (i) *node strength* as the sum of all weighted edges of connected nodes, indexing a parameter of network connectivity and connectivity strength; (ii) the *network core*, which is the largest subnetwork comprising all nodes with a degree of at least two that identifies the most tightly connected parts of a graph, providing insights into the overall structure of a network^44^; and (iii) *small-worldness* as the ratio of the normalized mean clustering coefficient (CC) and normalized characteristic path length (CPL). The CC calculates the proportion of existing clusters to all possible clusters. A cluster is defined as a triangle of connections between two connected neighbours of one node. The mean CC, reflecting the degree of functional specialization, is normalized to the average mean CC of 100 random networks with the same number of nodes, edges and degree distribution.^44^ The shortest path represents the smallest number of edges that connect two nodes. Here, we used the mean average of the shortest path length between all pairs of nodes in the network, normalized to a random network as described above. Complementary to CC, the CPL is used as a measure of integration within the network of interest. Crucially, small-worldness as the ratio of CC and CPL, with high values of CC and small values of CPL, enables both segregated and specialized but also distributed and integrated information processing. Thus, small-world topology is suggested to constitute an effective information processing structure, minimizing wiring cost while offering dynamical complexity.^7,46^ Finally, we calculated (iv) *modularity* by Newman’s spectral community detection algorithm,^44^ quantifying how much a network can be subdivided into nonoverlapping groups of nodes in a way that maximizes the number of within-modular edges and minimizes the number of between-modular edges. Consequently, modularity is supposed to represent the amount of functional specificity and information segregation within a network.^47^

### Statistical analysis

Statistical analyses were performed using the SPSS software package (Statistical Package for the Social Sciences, version 28, IBM). Group differences between stroke patients and healthy individuals were evaluated by independent two-sided *t*-tests (*P* < 0.05). In case of dynamic measures with multiple time periods, we performed repeated measures analyses of variance (5 levels: 0–50; 50–100; 100–200; 200–400; 400–600) × frequency (4 levels: DELTA; THETA; ALPHA; BETA) × group (2 levels: patients; controls).

Furthermore, Pearson’s correlations were computed to test for linear associations between behavioral scores and graph parameters. The behavioral scores were used as a motor composite score generated by a principal component analysis.^22^  *Post hoc* tests and correlation analyses were FDR-corrected for multiple comparisons.

## Results

### Increased integration of ipsilesional parietal areas early after stroke

**Figure 1:**
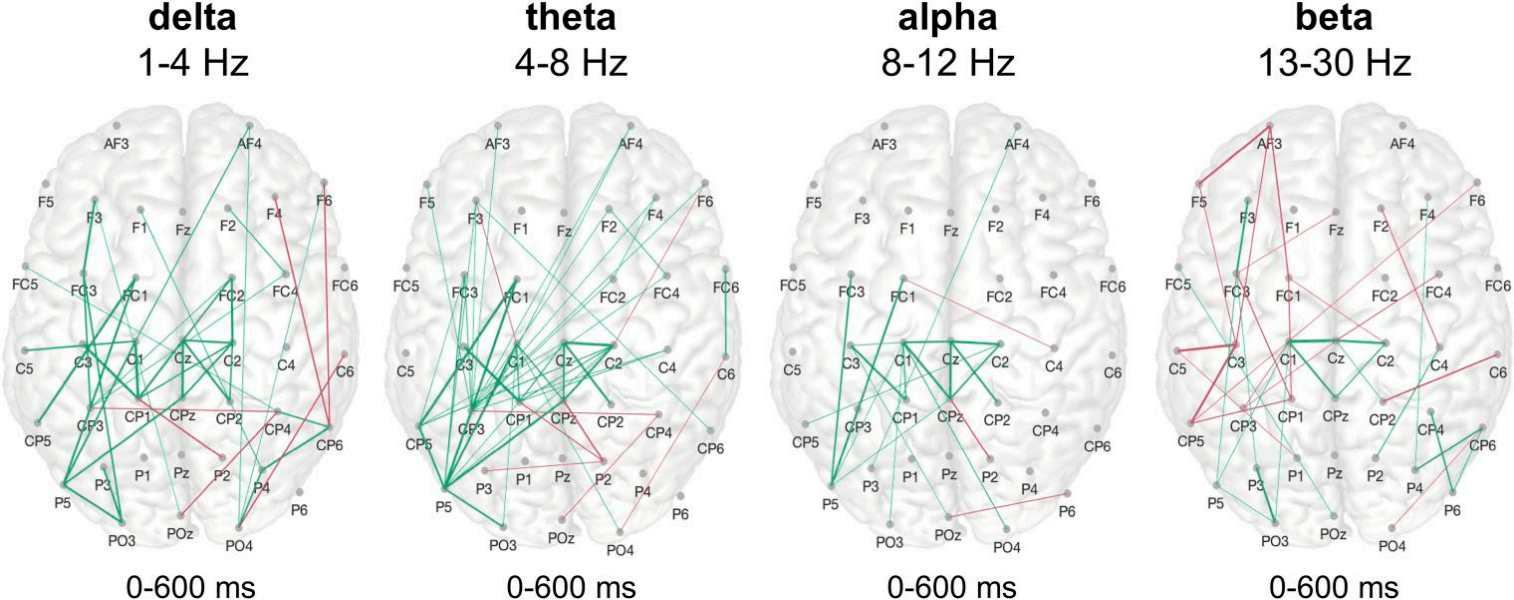
**Increase in low-frequency coupling in frontoparietal areas early after stroke.** The aggregated network connections over time post-stimulus indicated that patients in the first weeks after stroke showed an increase in coupling between frontocentral and parietal areas in the ipsilesional hemisphere, predominantly in the low-frequency delta and theta bands, but also in the alpha range, compared to healthy control subjects. For the beta frequency, stroke patients featured reduced coupling in the ipsilesional hemisphere, particularly between central and frontal regions. Please note that connections that appear more often are displayed with bolder graphs between sensors. The between-group comparison between healthy controls (*n* = 15) and stroke patients (*n* = 41) early after stroke is shown, illustrating the increase in slow oscillation coupling early after stroke (green: stroke > healthy participants; magenta: stroke < healthy participants, *P* < 0.05, FDR-corrected point-wise *t*-tests; left hemisphere = ipsilesional site, right hemisphere = contralesional site).

Analyzing the TMS-evoked connectivity, i.e. the PLV of TMS–EEG signals, in the first weeks after stroke, patients featured an increase in coupling between frontocentral and parietal areas in the ipsilesional hemisphere, particularly in the low-frequency delta and theta bands, but also in the alpha range, compared to healthy control subjects (*P* < 0.05, FDR-corrected, Fig. 1). Additionally, stroke patients showed enhanced coupling in response to the TMS perturbation in primary motor areas of both the ipsilesional and the contralesional hemisphere in the aforementioned spectra. Of note, for the beta frequency, stroke patients exhibited reduced connectivity in the ipsilesional hemisphere, especially between central and frontal regions (*P* < 0.05, FDR-corrected, Fig. 1).

**Figure 2:**
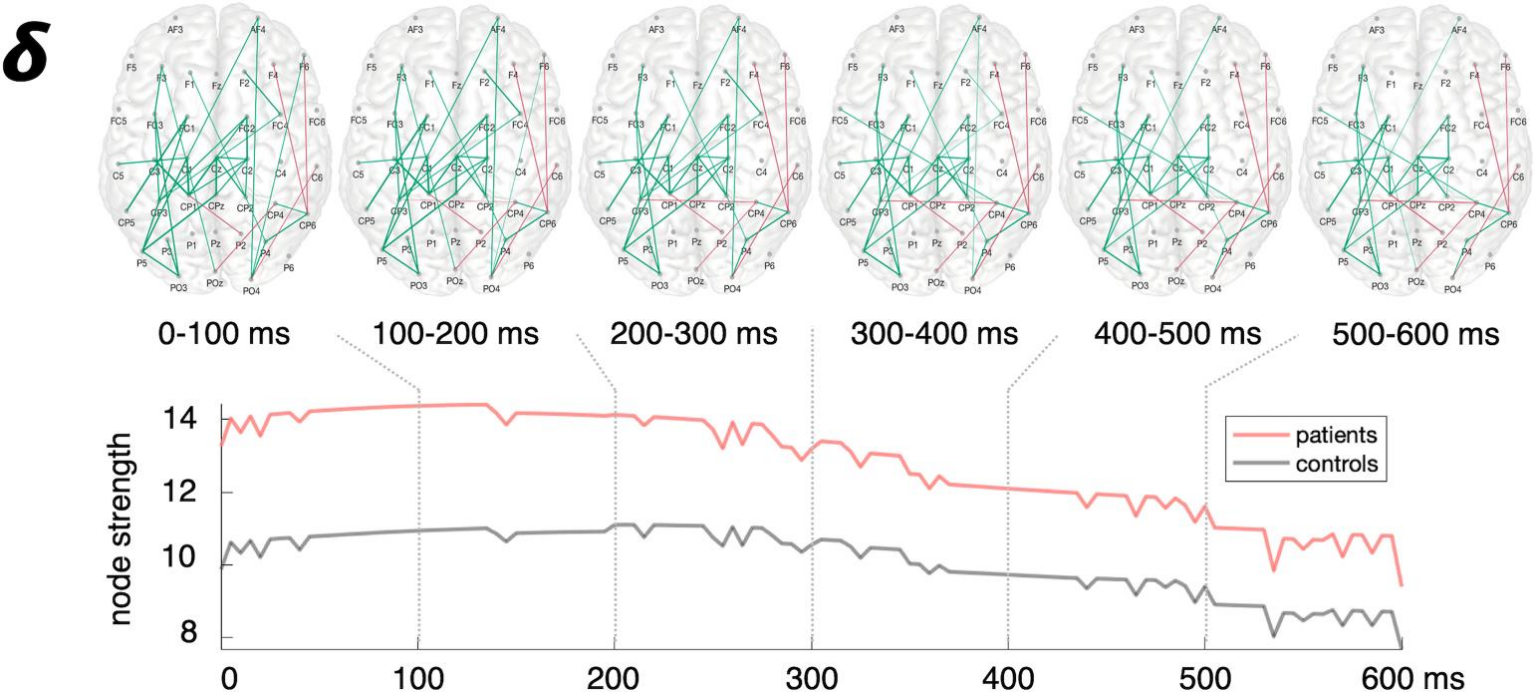
**Dynamic delta connectivity early post-stroke.** For the delta band, stroke patients showed increased coupling and, thus, increased node strength throughout the entire time period after TMS onset. Note that the upper panel displays the corresponding dynamic network connections along the post-stimulus period aggregated over the respective time period (colors indicate between-group comparison of connectivity with green: stroke (*n* = 41)>healthy participants (*n* = 15) and magenta: stroke < healthy participants, *P* < 0.05, FDR-corrected point-wise *t*-tests; left hemisphere = ipsilesional site, right hemisphere = contralesional site) and the lower panel shows the time course of overall node strength (red: patients, grey: control subjects). Please note that node strength was calculated on FDR-corrected between-group connectivity maps, eliminating non-significant time points and leading to a synchronization of the temporal progression. The dynamical analyses of the time course of node strength for each frequency band are depicted in Supplementary Fig. 2.

We next employed graph-theoretical analyses to further quantify the alterations in the network architecture post-stroke, especially in the temporal domain. Assessing the connection strength between all nodes in the network, analyses of the entire post-stimulus phase (0–600 ms) indicated an increase in node strength exclusively for the delta band early after stroke (delta: *P* = 0.03, *t*(50.4) = 2.70; theta: *P* = 0.18, *t*(54.0) = 1.71; alpha: *P* = 0.36, *t*(54.0) = 1.11; beta: *P* = 0.46, *t*(54.0) = 0.75; FDR-corrected). However, dynamically mapping the time course of node strength over the time after the TMS pulse (Fig. 2 and Supplementary Fig. 2) suggested differential trajectories. Accordingly, considering distinctive periods after TMS onset, we found significant between-group differences for node strength depending on the frequency band and the time post-stimulus, as revealed by a significant interaction effect for TIME × FREQUENCY × GROUP (*F*(12,648) = 2.04, *P* = 0.02) (main effect TIME: *F*(4,216) = 11.28, *P* < 0.001; main effect: FREQUENCY *F*(3,162) = 25.12, *P* < 0.001; interaction effect TIME × GROUP: *F*(4,216) = 2.78, *P* = 0.028; interaction effect FREQUENCY × GROUP: *F*(3,162) = 2.78, *P* = 0.096). A *post hoc* test revealed that early after stroke node strength was increased for all frequency bands within the first 50 ms after TMS onset compared to healthy controls (delta: *P* = 0.006, *t*(53.4) = 3.16; theta: *P* = 0.032, *t*(54.0) = 2.20; alpha: *P* = 0.007, *t*(52.0) = 2.82; beta: *P* = 0.006, *t*(53.8) = 3.24; FDR-corrected) (Supplementary Fig. 2). In the delta and theta range, the between-group differences lasted throughout the time period between 50 and 100 ms (delta: *P* = 0.014, *t*(53.8) = 3.03; theta: *P* = 0.014, *t*(33.4) = 2.93; alpha: *P* = 0.07, *t*(52.1) = 1.81; beta: *P* = 0.96, *t*(54.0) = −0.53; FDR-corrected). In contrast, after 100 ms the increase in node strength post-stroke was only significant in the delta band (delta: *P* = 0.024, *t*(54.0) = 2.84; theta: *P* = 0.38, *t*(54.0) = 1.33; alpha: *P* = 0.94, *t*(54.0) = 0.07; beta: *P* = 0.94, *t*(54.0) = 0.13; FDR-corrected) and remained enhanced here until 400 ms post-stimulus (delta: *P* = 0.028, *t*(51.3) = 2.54; theta: *P* = 0.51, *t*(54.0) = 0.88; alpha: *P* = 0.72, *t*(54.0) = 0.37; beta: *P* = 0.72, *t*(54.0) = −0.37; FDR-corrected; 400–600ms: all *P*-values ≥ 0.4) (Fig. 2).

Consequently, we focused on the delta and theta frequency bands for subsequent graph theoretical analyses. As implied by the differences in connectivity matrices (Fig. 1), the increase in node strength early after stroke was predominantly driven by coupling effects in the ipsilesional hemisphere (delta: *P* = 0.012, *t*(53.0) = 2.77; theta: *P* = 0.012, *t*(45.6) = 2.62; FDR-corrected), and here mainly by edges between frontocentral and parietal nodes (delta: *P* < 0.001, *t*(53.6) = 3.92; theta: *P* < 0.001, *t*(51.9) = 4.44; FDR-corrected). Notably, neither node strength of the contralesional hemisphere nor of ipsilesional frontal nodes showed significant differences between patients early after stroke and healthy participants (contralateral nodes: delta: *P* = 0.16, *t*(54.0) = 2.08; theta: *P* = 0.16, *t*(54.0) = 1.77; ipsilesional frontal nodes: delta: *P* = 0.79, *t*(54.0) = 0.27; theta: *P* = 0.53, *t*(54.0) = 0.85), underlining the region specificity of the findings.

### The network architecture post-stroke and reduced efficiency of information flow

**Figure 3:**
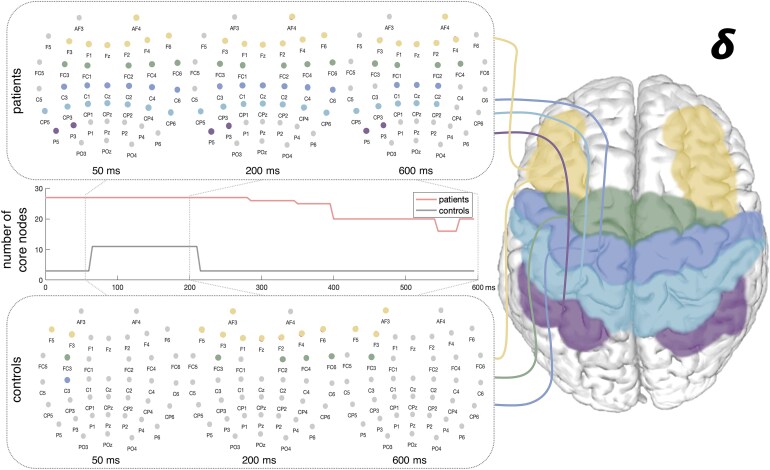
**Stroke is associated with a broader network core comprising bihemispheric centroparietal and ipsilesional parietal nodes.** The significant core nodes over the time post-stimulus revealed an extensive bihemispheric network topology that lasted up to 600 ms post-stimulus in the delta early after stroke. The upper panel shows the network core of stroke patients in comparison to the network of healthy controls in the lower panel. In the middle, the time course of significant core nodes for the entire post-stimulus period is depicted (between-group comparison *t*-test: stroke (*n* = 41) and healthy participants (*n* = 15); delta: *P* = 0.011, *t*(54.0) = 2.77; FDR-corrected; left hemisphere = ipsilesional site, right hemisphere = contralesional site). The analyses of significant core nodes for the delta and theta frequencies are shown in Supplementary Fig. 3.

Complementary, global network properties were also altered early after stroke. TMS-evoked interregional coupling exhibited a spatially broader network core in the low-frequency range of delta and theta in stroke compared to healthy controls (delta: *P* = 0.011, *t*(54.0) = 2.77; theta: *P* = 0.011, *t*(16.9) = 2.85; FDR-corrected), i.e. the largest subnetwork comprising all nodes with a degree of at least two.^45^ Portraying the significant core nodes over time post-stimulus illustrated an extensive bihemispheric network topology that lasted up to 600 ms post-stimulus (Fig. 3 and Supplementary Fig. 3). In stroke patients, TMS-induced coupling formed a core of nodes comprising bihemispheric centroparietal and ipsilesional parietal electrodes over the entire post-stimulus period, especially in the delta frequency band, while in healthy controls, the core nodes recruited by TMS predominantly involved frontal and frontocentral electrodes.

However, the architecture of networks cannot only be characterized by the involved nodes and connection strengths in-between. The complex network organization of the brain holds properties of modularity and small-world topology, promoting efficient information flow and segregation of information for resilience. Accordingly, in the first weeks after stroke, the network evoked by TMS to the ipsilesional M1 featured a substantial decrease in small-worldness in the delta range (delta: *P* = 0.016, *t*(54.0) = −2.74; theta: *P* = 0.62, *t*(54.0) = 0.31; FDR-corrected). Crucially, the decrease in small-worldness was predominantly caused by increased path length (*P* = 0.009, *t*(50.4) = 2.70). Thus, early after stroke, signal propagation from specialized brain regions requires considerably more processing steps for information integration and, thus, substantially higher costs in terms of information flow efficiency.

Furthermore, we found a reduction in modularity again for the delta frequency (delta: *P* = 0.038, *t*(30.8) = −2.17; theta: *P* = 0.17, *t*(54.0) = −1.38; FDR-corrected), implying a deterioration of the organization in functionally specific subnetworks and, thereby, a decrease in information segregation early after stroke.

Notably, while alterations in node strength were mainly attributed to the ipsilesional hemisphere, stroke-associated changes in the higher network structure were found in both the ipsilesional and the contralesional hemisphere (small-worldness: ipsilateral: *P* < 0.001, *t*(54.0) = −3.54, contralateral: *P* = 0.031, *t*(54.0) = −2.22; modularity: ipsilateral: *P* = 0.025, *t*(34.8) = −2.52, contralateral: *P* = 0.025, *t*(54.0) = −2.43; FDR-corrected).

### Bifrontal connectivity and motor deficits early after stroke

Despite the profound alterations in the network configuration in the first 2 weeks after stroke, there was no association between the observed changes in the low-frequency bands and the clinical deficit assessed for the whole group of patients in the early phase after stroke, i.e. NIHSS and motor scores (all *P*-values > 0.2). Thus, the parietal shift in TMS-induced coupling and the reduction of a modular and small-world network topology in the delta frequency seem to be a general phenomenon rather than linked explicitly to the acute clinical deficit.

To further explore network dynamics underlying motor function after stroke, we divided the patient group featuring a broad range of clinical deficits (grip strength: 28.2 ± 35.7%; 0–94.7%; ARAT: 18.1 ± 22.7; 0–55; MI: 42.5 ± 34.0; 0–95.5) into two subgroups using k-means cluster analysis based on the acute motor deficit indexed by the composite score. The resulting groups represented a subgroup of severely affected patients (*n* = 25; grip strength: 2.0 ± 6.9%; 0–29.3%; ARAT: 0.9 ± 1.5; 0–3; MI: 18.1 ± 15.8; 0–58) and a subgroup of patients with mild to moderate motor deficits (*n* = 16; grip strength: 69.2 ± 19.9%; 43.5–94.7%; ARAT: 44.9 ± 10.2; 27–55; MI: 80.5 ± 12.7; 47–95.5).

**Figure 4:**
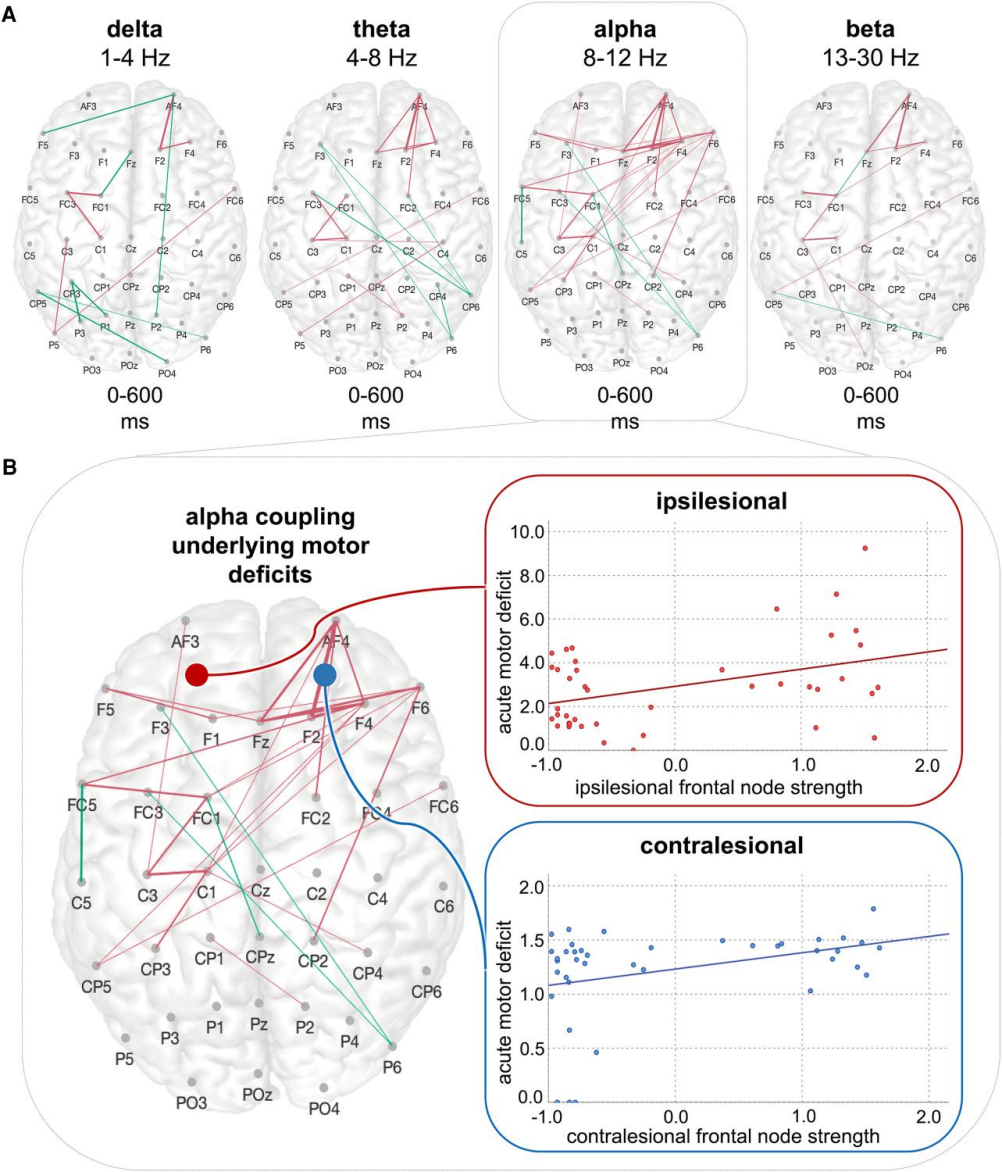
**Bifrontal connections are indicative of the motor deficit early after stroke.** (**A**) Between-group comparisons between the subgroups of severely affected patients and mildly to moderately affected patients indicated a decrease in node strength in more affected patients in the alpha band within ipsilesional and contralesional frontal areas (green: severe > moderate to mild and magenta: severe < moderate to mild, *P* < 0.05, FDR-corrected point-wise *t*-tests; left hemisphere = ipsilesional site, right hemisphere = contralesional site). (**B**) Alpha node strength of frontal connections in both the ipsilesional and the contralesional hemisphere was linked to the acute motor deficit. Please note that single data points represent individual patients (*n* = 41) with their individual level of acute motor deficit and strength of the frontal connection of the respective hemisphere (Pearson’s correlation: ipsilesional: *r* = 0.41, *P* = 0.016; contralesional: *r* = 0.35, *P* = 0.025; FDR-corrected).

Contrasting the TMS-evoked coupling effects between the two subgroups of patients, i.e. severely affected patients and moderately to mildly affected patients, revealed that severely affected patients featured a reduction in contralesional frontal connections, particularly in the alpha frequency but also in the theta and beta range (*P* < 0.05, FDR-corrected, Fig. 4A). Likewise, decreased frontal coupling was also found in the ipsilesional hemisphere in the alpha band.

Correspondingly, between-group comparisons indicated a decrease in node strength in severely affected patients compared to mildly to moderately impaired patients in the alpha band within ipsilesional and contralesional frontal areas (ipsilesional: delta: *P* = 0.22, *t*(39.0) = −1.36; theta: *P* = 0.09, *t*(39.0) = −2.09; alpha: *P* = 0.016, *t*(39.0) =−3.09; beta: *P* = 0.22, *t*(22.1) = −1.26; contralesional: delta: *P* = 0.75, *t*(39.0) = −0.32; theta: *P* = 0.53, *t*(39.0) =−0.91; alpha: *P* = 0.04, *t*(32.5) = −2.74; beta: *P* = 0.53, *t*(39.0) = −0.85; FDR-corrected). Beyond that, alpha node strength of frontal connections in the ipsilesional and the contralesional hemisphere was associated with the composite score of the acute motor deficit (ipsilesional: r = 0.41, *P* = 0.016; contralesional: r = 0.35, *P* = 0.025; FDR-corrected), indicating that stronger TMS-evoked bifrontal alpha coupling underlies better motor function early after stroke (Fig. 4B).

### Reorganization of TMS-evoked coupling and recovery post-stroke

**Figure 5:**
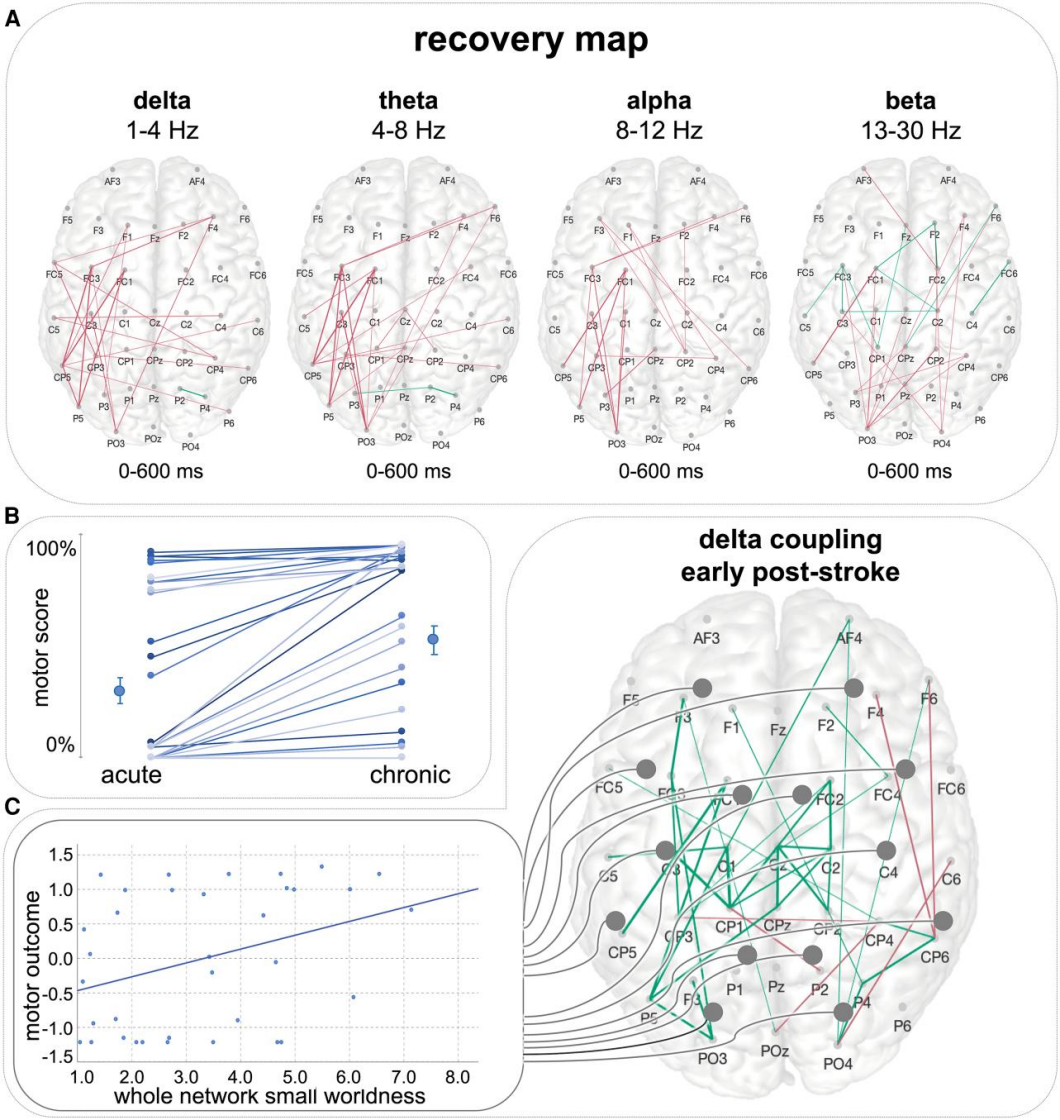
**Motor recovery after stroke is paralleled by a normalization of increased delta coupling.** (**A**) Network connections aggregated over time post-stimulus for within-group comparison of stroke patients early after stroke and in the chronic post-stroke phase (magenta: acute > chronic; *P* < 0.05, FDR-corrected point-wise *t*-tests; left hemisphere = ipsilesional site, right hemisphere = contralesional site). (**B**) Motor recovery and motor reorganization were paralleled by a decrease in the initially augmented coupling in parietofrontal areas in the low-frequency range. Single data points represent the individual trajectories of the functional deficit of each patient from the acute to the chronic stage post-stroke. (**C**) By contrast, the decrease in small-worldness early post-stroke in the delta range was indicative of a poor motor outcome. Here, single data points depict individual patients (*n* = 41) with their individual level of motor outcome and the extent of alteration of small-worldness early post-stroke (Pearson’s correlation: *r* = 0.37, *P* = 0.029).

In the follow-up session post-stroke, stroke patients showed substantial motor recovery (*P* < 0.001, *t*(34.0) = −4.80) (Fig. 5B). In parallel, the connectivity map of reorganization, i.e. the significant group differences in coupling aggregated over time post-stimulus between the 3 months of follow-up and the first weeks post-stroke, revealed a decrease in connections, particularly between ipsilesional frontocentral and parietal areas in the low-frequency range of delta, theta and alpha (all *P* < 0.05, FDR-corrected, Fig. 5A).

Here, node strength of frontoparietal coupling in the ipsilesional hemisphere, which was increased in comparison to healthy participants in the first weeks post-stroke in the delta and theta frequency, significantly decreased in these frequency bands and also in the alpha band (delta: *P* = 0.034, *t*(65.9) =1.86; theta: *P* = 0.026, *t*(67.0) = 2.30; alpha: *P* = 0.026, *t*(67.0) = 2.16; FDR-corrected) during motor reorganization.

Along with this normalization of the initially enhanced interregional coupling in low-frequency spectra, characteristics of the complex network structure further strived to reinstate. Correspondingly, the reduction in small-worldness in the delta frequency found early post-stroke had improved after >3 months after stroke (*P* = 0.029, *t*(55.8) = −1.93). Likewise, we found a significant increase in modularity in the delta frequency at follow-up 3 months later (*P* =0.036, *t*(66.7) = −1.83). Thus, properties of complex network organization, which were substantially impaired early after stroke, demonstrated an amelioration in parallel to motor recovery.

Finally and crucially, the acute decrease in small-worldness early after stroke was linked to the motor outcome after >3 months post-stroke (r = 0.37, *P* = 0.029). Thus, patients featuring a particular decrease in small-world topology in the delta band showed a less favourable motor outcome (Fig. 5C).

## Discussion

Combining TMS–EEG, dynamic connectivity analyses and graph theory, we draw a link between an increase in low-frequency coupling in the delta band and alterations in the complex network topology early after stroke and their functional relevance for motor recovery in the chronic post-stroke phase. Specifically, we demonstrated that stroke is associated with increased slow activity and that stroke lesions induce enhanced delta coupling between frontocentral and parietal regions, replicating the findings of parietal involvement in fMRI studies.^17,18^ However, at the level of the higher network structure, we also found a loss of the physiological network architecture with a decrease in small-worldness and modularity in the delta frequency band, implying a stroke-associated alteration of both information integration and segregation within the first weeks after stroke. While bifrontal connections in the alpha spectrum seem to be crucial for the functional deficit in the early post-acute phase, solely the impairment of small-worldness in the delta frequency indicated a more pronounced permanent deficit. By contrast, after >3 months post-stroke, we found a normalization of increased low-frequency coupling and a restitution of the complex network structure featuring a small-world and modular topology in parallel to motor recovery.

### Connectivity, randomness and diaschisis after stroke

The finding of stroke-induced changes in functional and effective connectivity of the intra- and interhemispheric network architecture has been substantiated by fMRI studies (see, e.g. Grefkes and Fink^15^ or Siegel *et al*.^48^ for review) and aligns with our TMS–EEG data. Accordingly, reorganization of the motor network after stroke encompasses not only an acute reduction in interhemispheric connections and connectivity between ipsilesional M1 and frontoparietal regions very early after stroke but also a gradual increase and restoration of network interactions in the following weeks and months.^49-51^ Furthermore, neural networks reorganizing post-stroke showed increased facilitatory coupling between M1 and frontoparietal areas in the affected hemisphere.^52,53^ In particular, most analyses have focused on frontal motor circuits, comprising the premotor cortex and supplementary motor area, yet there is also evidence from fMRI studies for increased connectivity with the ipsilesional parietal cortex in stroke patients, paralleling the present results.^50,54,55^ As the reconstitution of physiological connectivity patterns has been associated with a favourable recovery in the chronic phase, these network alterations are suggested to be functionally relevant.^15,53^ However, the body of fMRI connectivity studies has still not elucidated whether changes in connectivity enable functional recovery or merely represent the neuronal aftermaths of adaptation and compensation. For parietofrontal connections, both enhanced fMRI coupling within the first weeks post-stroke and increased connectivity strength in the chronic phase were associated with a less favourable chronic outcome and, thus, a more severe permanent impairment.^54,55^ This upregulation has been interpreted as a manifestation of compensation and additional recruitment of parietal areas involved in sensory processing and sensorimotor integration.^56^ Although we found a comparable enhancement of coupling between frontocentral and parietal regions, the unique methodological combination of TMS–EEG, connectivity and graph theory will, however, dispute the previous understanding. In particular, by linking increased frontoparietal coupling early after stroke to the loss of the physiological network architecture, our data rather question the compensatory nature.

Accordingly, beyond investigating changes in specific functional subnetworks and away from descriptive anatomy towards measures of network efficiency and functionality, the application of graph theory on brain connectivity data allows to disclose the lesion’s impact on the global network organization and its complex relationship within and between communities of highly connected nodes promoting functional segregation while preserving functional integration, thus, potentially reflecting the complexity of behavioral deficits more accurately.^7,57^ The small-world organization is essential for the resilience of the brain architecture and for protecting its network integrity.^58^ In an experimental stroke model, increased small-worldness of functional connectivity in the somatosensory network was found in the subacute phase, suggesting to reflect excessive neuronal re-wiring, which shifted to baseline topology with recovery of sensorimotor functions in the chronic phase.^51^ The finding of increased small-world topology is underpinned by a translational approach with a comparable pattern of network parameters in the early subacute phase in mildly affected mice and humans, indicating a facilitation of the global communication structure.^19^ In contrast, other fMRI studies have reported a decrease in small-world topology after stroke, especially in more severely affected patients,^12,17^ which underlines our present findings. Accordingly, in a longitudinal study of subcortical strokes leading to severe upper limb deficits, the motor network gradually shifted towards a random mode with time after stroke during the recovery process and in association with impairment, suggesting that a less optimized network reorganization is involved in regaining motor function.^12^ Furthermore, and similarly to the present results, Siegel and colleagues found a decrease in small-worldness and modularity already in the early post-acute phase,^17^ implying that both integration within and segregation between the functional networks are reduced early after stroke. Again, in line with our results, the return of a modular network featuring a small-world topology partially recovered over time and in parallel to functional recovery.^17^ Complementary evidence also stems from EEG studies that revealed a frequency-dependent modulation of the small-world properties with an increase in small-worldness in the alpha band but a stroke-associated decrease in the delta frequency.^23^ The most parsimonious explanation for these primarily contradictory results is that damage to brain regions critical for information processing *within* a particular subdomain may affect the functional segregation of neural networks, while a lesion to a region engaged in communication *between* subdomains was found to impact whole-brain network organization.^59-61^

A shift towards network randomization was also shown for other neuropathologies like brain tumour, severe traumatic brain injury, or Alzheimer’s disease, implying a common brain response to different lesion types and processes.^62-65^ Although the underlying substrates for network randomization remain speculative, potential explanations might lie in a compensatory but non-optimized outgrowth of new connections because of structural or functional disconnection of physiological pathways, modulation of synaptic activity in the networks, alterations in the timing of information transfer or the unmasking of silent pathways as a consequence of deafferentation.^51,62,66^ Along these lines, alterations in the network dynamics, more precisely, a shift towards randomization, also have to be considered a manifestation of diaschisis, revealing a non-optimized architecture. To be recognized as diaschisis, alterations ought to be most pronounced early after stroke and progressively normalize over time, in parallel to function and behavior.^14^ However, whether these alterations in the network structure after stroke arise either in the context of diaschisis or from plasticity mechanisms beneficial for functional recovery after stroke have yet remains unsolved. Here, the methodology of TMS–EEG may offer a unique combination of methodological characteristics, allowing for an unrivalled activation of the motor system, delineating its functional relevance,^62^ assessing effective connectivity by dissolving temporal dynamics and causal interactions between the stimulated area and subsequent regions, and incorporating oscillatory properties holding functional information, thereby revealing pathophysiological mechanisms.

Accordingly, at the level of anatomical subnetworks, we demonstrated an increased coupling within parietofrontal circuits early after stroke, which subsequently and progressively decreased with time post-stroke. At the level of the global network architecture, we found a decrease in modularity and small-worldness in the first weeks after stroke, followed by a restoration of the physiological network architecture after >3 months post-stroke, along with functional improvement. In the dimension of oscillations, these alterations were predominantly evident for slow rhythms, particularly in the delta frequency. Crucially, the decrease in small-worldness in the delta band was associated with the functional outcome in the chronic stroke phase, with initially more pronounced alterations in the network architecture leading to a less favorable outcome. In summary, the observed stroke-induced connectivity changes in the slow frequency range need to be understood in the context of diaschisis as defined above, implying a link between diaschisis, network randomization, the familiar phenomenon of a stroke-induced EEG slowing, and the functional implications of delta activity.

### Linking increased slow activity early after stroke to network randomization and post-stroke recovery

An increase in slow activity particularly in the perilesional cortex but also in areas distant from the lesion within the unaffected hemisphere has been described as the predominant finding in early EEG recordings post-stroke almost half a century ago.^21,26^ Ever since, enhanced activity of slow oscillations, characteristically quantified as low-frequency power, has been detected in animal models^67-69^ and patient studies from the acute to the chronic stroke phase.^70,71^ In the ipsilesional hemisphere, augmented delta activity has often been found to be accompanied by a depression of faster oscillations, i.e. in the alpha and beta frequency bands.^25,72,73^ From a clinical perspective, alone or in combination with an ipsilesional alpha power decrease, an ipsi- and contralesional EEG slowing has been associated with a poor outcome post-stroke.^70^ More mechanistically, delta waves were shown to be related to stroke-induced ischemia and tissue dysfunction.^74^

However, although the mechanisms underlying physiological slow activity have been distinctly identified, the neural underpinnings of stroke-associated slow oscillations remain to be determined. Notably, in this context, the terms of slow activity, indicating the faster spectrum of delta power, and sleep-like slow waves, referring to slow oscillatory activity <1 Hz as the manifestation of off-periods and cortical bistability,^75-77^ are not always unambiguously separable in the literature, although recent work has demonstrated different regulation principles and, thus, potentially distinct mechanistic circuits.^78^ While the occurrence of sleep-like slow waves has been recently demonstrated to happen at the local level in stroke patients,^22,24^ the present work is rather signified to slow activity in the delta range from 1 to 4 Hz. While sleep-like slow waves can be cortically generated without thalamic contribution,^79,80^ delta waves are primarily generated and modulated by thalamocortical circuitries.^81^ Accordingly, thalamocortical relay neurons project to the cortex and, in a hyperpolarized voltage range, hold an intrinsic burst-firing activity, constituting the generator of cortical delta waves.^82,83^ This self-oscillatory activity arises from the orchestrated interplay between hyperpolarization-activated cyclic nucleotide-gated (HCN) channels and T-type voltage-gated calcium (CaV) channels, and rapidly deactivating voltage-sensitive potassium channels.^82,84-86^ However, since thalamocortical neurons only generate delta waves in hyperpolarization and burst-firing mode, inhibitory inputs are crucial for initializing and promoting delta waves, resulting from inhibitory GABAergic input of the thalamic reticular nucleus.^81,87^ Crucially, in this context, HCN and CaV channels have been shown to be involved in the pathology of ischemic brain injury.^88,89^ Moreover, there is evidence for increased tonic inhibition governed by extra-synaptic GABAA receptors after stroke.^90^ As pharmacological studies revealed that under physiological conditions, enhancing the GABAergic influence on thalamocortical neurons mediated through extra-synaptic GABAA receptors enhances delta waves,^91-93^ similar neuronal mechanisms determined for non-rapid eye movement sleep (NREM) delta waves may equally apply to the underpinnings of increased slow activity after stroke. This appears particularly relevant in view of the contemporary understanding that sleep does not necessarily occur exclusively at the behavioral level as a distinct global state, but also during wakefulness locally confined to discrete cortical areas with detrimental behavioral consequences,^24,94,95^ again opening a parallel to slow activity after stroke and stroke-induced functional deficits.

Likewise, the deceleration of alpha power post-stroke,^25,72,73^ accompanied by increased slow activity, is also well explained as alpha rhythms are strongly influenced by the thalamus and underlie the same cellular components of thalamocortical relay neurons outlined above, albeit under depolarized conditions, for example, when activated by cortical glutamatergic input.^96,97^ One might speculate that alterations in thalamocortical network dynamics associated with an attenuated neocortical drive onto the thalamus, i.e. deafferentation and, thus, thalamic disfacilitation and pathologically high levels of inhibition, potentially as found in stroke, might lead to hyperpolarization and, thereby, to both the depression of alpha activity and the increase in slower frequencies.^72,96^

Notably, as argued above, we interpret the increase in frontoparietal coupling and the alteration in the functional network architecture, i.e. the network randomization, early after stroke in terms of diaschisis. Moreover, we found these changes exclusively in the slow frequency range. Thus, we suggest that connectional diaschisis after stroke might be related to the disruption of thalamocortical circuits. In other words, pathological lesion-induced thalamocortical dynamics propagate low-frequency oscillations beyond the lesion through thalamocortical pathways to widespread areas of the neocortex.^72^ The resulting connections form an overconnected, random network that lacks a resilient and optimized small-world topology, similar to what we found in the present study. Admittedly, without the verification of an alteration in the thalamocortical circuit, arguments remain indirect and speculative. However, in accordance with our findings, inactivation of a cortical node by chemogenetic inhibition led to increased connectivity between the inhibited area and its direct thalamocortical target in an animal model.^98^ Using *in vivo* electrophysiology, the authors could also show an enhancement of low-frequency oscillations via the suppression of neural firing, resulting in increased delta coherence between regions that exhibited increased coupling,^98^ resembling our findings of enhanced coupling in the delta range early after stroke. Experimental recordings from deafferented cortical slabs isolated from thalamic and cortical inputs have revealed an amplification of slow oscillations, underlining the link between disconnection and slow activity,^80^ which is particularly relevant in the context of stroke. Furthermore, the thalamus and thalamocortical connections in the affected hemisphere have been shown to play a crucial role in neuronal reorganization and functional recovery after stroke.^99^ Moreover, optogenetic stimulation of ipsilesional M1 in a stroke mice model restored activation in the ipsilesional corticothalamic circuit and improved functional recovery, stressing the involvement of corticothalamic loops in recovery after stroke.^100^

Beyond the potentially underlying thalamocortical mechanisms, other complementary explanations in the development of stroke-associated slow activity, such as cortical deafferentation and cortico-cortical slow wave propagation, have to be considered.^22,101^ For example, cortical deafferentation and damage to white matter fibres induced slow waves due to a loss of input signal from ascending activating systems due to damage of white matter fibres.^68^ Yet, using a neurophysiological corticothalamic circuit model, a very recent study showed that slowing after stroke is indeed mediated by thalamic disinhibition, resulting from decreased input from the inhibitory thalamic reticular nucleus rather than from alterations in the cortex or white matter connections.^102^

Along these lines, one might argue that the absence of spindles speaks against a major contribution of the thalamus to the observed alterations after stroke. However, several sources might cause this phenomenon without fundamentally questioning the involvement of the thalamus in post-stroke slow oscillations. Accordingly, in epilepsy, it has been shown that thalamic spiking inhibits the occurrence of spindles, leading to impaired cognitive function.^103^ Moreover, depriving thalamic neurons of the nucleus reticularis abolished spindle oscillation in an animal model,^104,105^ paralleling the decreased input from the inhibitory thalamic reticular nucleus after stroke.^102^

Our data indicate that impairment of the complex network structure in the low-frequency range was especially related to a less favourable outcome. Delta waves, as found under physiological conditions, seem to be critically implicated in many processes that benefit memory, cognition, synaptic homeostasis, cellular energy regulation and neuronal plasticity.^106-109^ Hence, enhanced TMS-induced activity in slow frequency bands in awake stroke patients might be understood within the context of neural mechanisms engaged in plasticity and compensatory re-wiring during reorganization. However, given that patients showing enhanced delta coupling featured alteration of both information integration and segregation, i.e. small-worldness and modularity, and a poorer outcome, and given that slow activity may result from disrupted thalamocortical activity and imbalances in thalamocortical inhibition, it might also constitute a process interfering with successful reorganization.

## Conclusion

We here identified increased frontoparietal low-frequency coupling and the deterioration of a physiological network architecture early after stroke in the context of diaschisis. By adding the functional layer of oscillatory properties to connectivity, we revise the current understanding of the neural substrate of diaschisis and remote effects of focal lesions by associating them with a disturbance of thalamocortical circuits. As we did not record individual treatment protocols, we could not rule out the impact of different rehabilitation regimes. However, our findings also motivate novel treatment strategies for post-stroke deficits and neuromodulatory interventions, particularly suggesting subcortical structures as a target for therapy and demanding techniques capable of reaching these deeper regions.^110,111^

## Supplementary Material

fcaf391_Supplementary_Data

## Data Availability

The data and codes that support the findings of this study are available from the corresponding author (C.G.) upon reasonable request.
