# Supplementary material for: Evoked slow oscillations and dynamic network reorganization after stroke
Source: Brain Commun. 2025 Oct 16;7(6):fcaf391. doi: 10.1093/braincomms/fcaf391 (PMC12576543; doi:10.1093/braincomms/fcaf391)
Supplement: fcaf391_Supplementary_Data [file fcaf391_supplementary_data.docx]

**SUPPLEMENTARY MATERIAL**

1. TMS procedure

Primary motor cortex (M1) was defined by the motor hotspot, i.e., the position of the TMS coil held tangentially to the skull in a 45° posterior-anterior direction eliciting motor evoked potentials (MEP) of the highest amplitudes of the first interosseous (FDI) muscle. Ag/AgCl self-adhesive electrodes were placed over the muscle according to the belly-tendon montage. The EMG signal was amplified, high- and band-pass filtered (0.5Hz high-pass; 30-300Hz band-pass), and digitized using a Powerlab 26T device combined with the LabChart software (Package version 8.0; AD Instruments, Sydney, Australia).

TMS was controlled by a frameless computerized stereotactic neuronavigation system throughout the experiment (BrainSight V.2.0.7; Rogue Research Ltd; Montreal, Canada). For this purpose, the participants’ heads were co-registered with an individual anatomical MR image which allowed to reliably monitor online the position of the TMS coil in reference to the stimulation coordinated and the subject’s head, within and across sessions.

1. TMS-EEG recordings

TMS-EEG was recorded using a 64-channel TMS-compatible EEG system (BrainAmp DC, BrainProducts GmbH, Gilching, Germany) that prevents amplifier saturation and reduces the induced magnetic artifacts. The EEG signals were sampled at 5kHz with a resolution of 0.1µV per bit, high-pass filtered with 0.1Hz, and low-pass filtered with 1kHz. Scalp EEG was recorded by 62 Ag/AgCl sintered ring electrodes mounted on an elastic electrode cap (EasyCap) following the international 10-20 system. Horizontal and vertical eye movements were recorded using the two remaining electrodes as electrooculogram (EOG) sensors. Impedances of all electrodes were kept below 5kΩ throughout the whole experiment, a crucial prerequisite to minimize artifacts. Participants wore inserted earplugs to reduce EEG auditory evoked potentials induced by the TMS click.^1–3^ Masking the TMS click with white noise^4^ has proven difficult in acute stroke patients due to the high volume levels needed for sufficient masking (>90 dB). Using the former approach, we have previously shown to capture a biologically relevant neural signal largely without significant confounds of peripheral co-activation.^5^ Moreover, bone conduction produced by TMS was attenuated by placing a thin layer of film between the coil and EEG cap.^5–7^

Subjects were seated in a comfortable chair with eyes open during the EEG recordings. Severely affected stroke patients, who were unable to sit due to their deficit, were assessed in their beds with a 45° incline of the headboard (n=23).

For each TMS-EEG session, we collected at least 100 trials of single pulses randomly jittered between 6.5-8.0s and with an intensity of 80% RMT. This intensity range is considered to be above the threshold for a significant EEG response, but still minimizes re-afferent somatosensory feedback impacting on the EEG response.^5,8–11^

1. TMS-EEG analysis

Data analysis was performed using Matlab (v2019; The MathWorks, Massachusetts, USA). Channels and trials that were contaminated by artifacts were visually inspected and manually rejected.^5,12,13^ Recordings with either less than 90 artifact-free trials or more than 10 poor channels were excluded from further analysis. Data within -2ms and 10ms relative to TMS pulse onset were removed and missing data were replaced with baseline.^5,14^ Subsequently, EEG data were detrended, band-pass and band-stop filtered (1-60Hz; 49-51Hz; Butterworth 3^rd^ order), down-sampled to 625Hz, and segmented in time windows of ±1000ms around the TMS pulse.^5,13^ Bad channels were interpolated using the EEGLAB spherical interpolation function (<https://sccn.ucsd.edu/eeglab/>)^15^. Signals were average re-referenced and baseline-corrected.^13^ Finally, independent component analysis (ICA, EEGLAB runica function) was applied to remove residual ocular, muscle, or TMS-related artifacts.^16^

1. Calculation of PLV connectivity

To minimize the effect of volume conduction, preprocessed epochs were re-referenced to the Laplacian reference (<http://psychophysiology.cpmc.columbia.edu/Software/CSDtoolbox>) to improve spatial resolution and the suitability for subsequent connectivity analysis.^17,18^

The time-frequency decomposition, which allowed to compute the temporal evolution of amplitude and phase for each frequency separately in the delta- to beta-frequency range (1-30Hz), was performed using the Statistical Parametric Mapping toolbox (SPM12; The Wellcome Centre for Human Neuroimaging, London, UK, <http://www.fil.ion.ucl.ac.uk>) implemented in Matlab.

The coupling between two brain regions was calculated by the synchronization between the activity of two sites as defined by the single-frequency phase-locking value at the sensor level (PLV) ^19-21^, resulting in a positive number between 0 and 1 with PLV=1 occuring in cases of perfect intertrial phase locking of the phase difference between the EEG signals of a pair of two channels over all trials. We, here, considered the four frequency bands: δ (1-4Hz), θ (4-8Hz), α (8-12Hz) and β (13-30Hz). We calculated the PLV separately for each frequency with a resolution of 1Hz and subsequently averaged the values over the respective frequency band.^21^ This analysis yielded 760 connectivity values for each frequency and each participant.

To test for significance of PLV values obtained for each pair of electrodes at each time point of the post-stimulus interval of interest, i.e., 0-600ms, we used a pointwise t-test with a significance level of p<0.05 FDR-corrected for multiple comparisons. The corrections were performed concerning the number of time points, groups, and electrodes.^21^

**Supplementary Figure 1 – Lesion overlap of stroke patients**


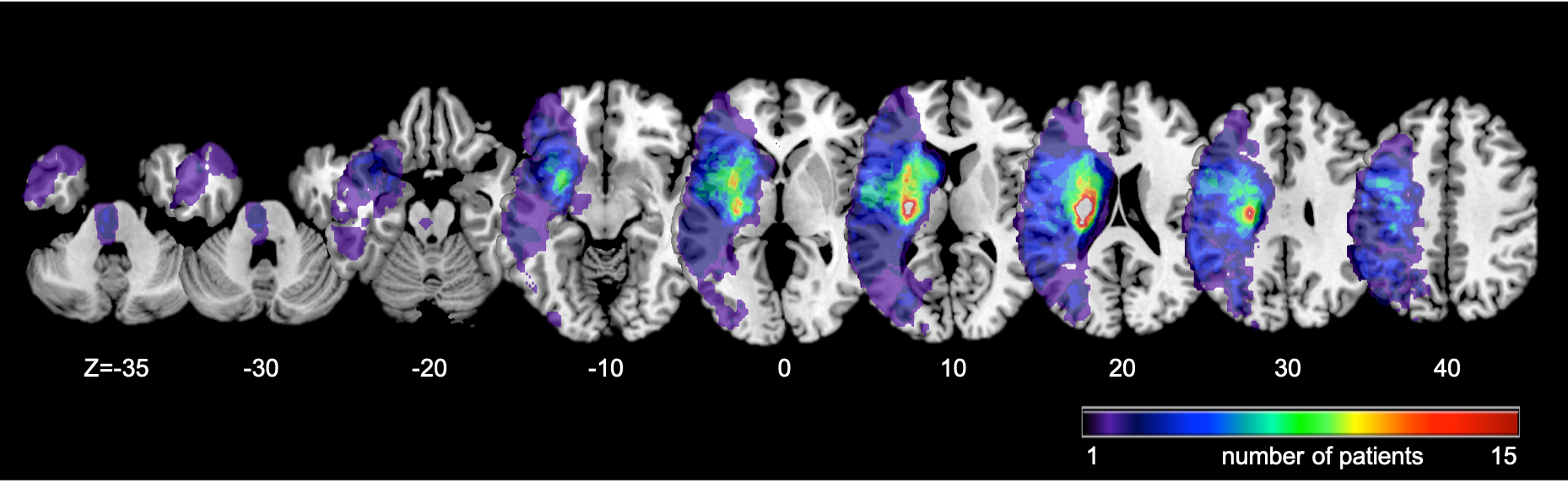


Lesion overlap of stroke patients based on individual MR images. Stroke patients showed the maximum overlap at the level of the posterior limb of the internal capsule.

**Supplementary Figure 2 – Dynamic connectivity early post-stroke**

**
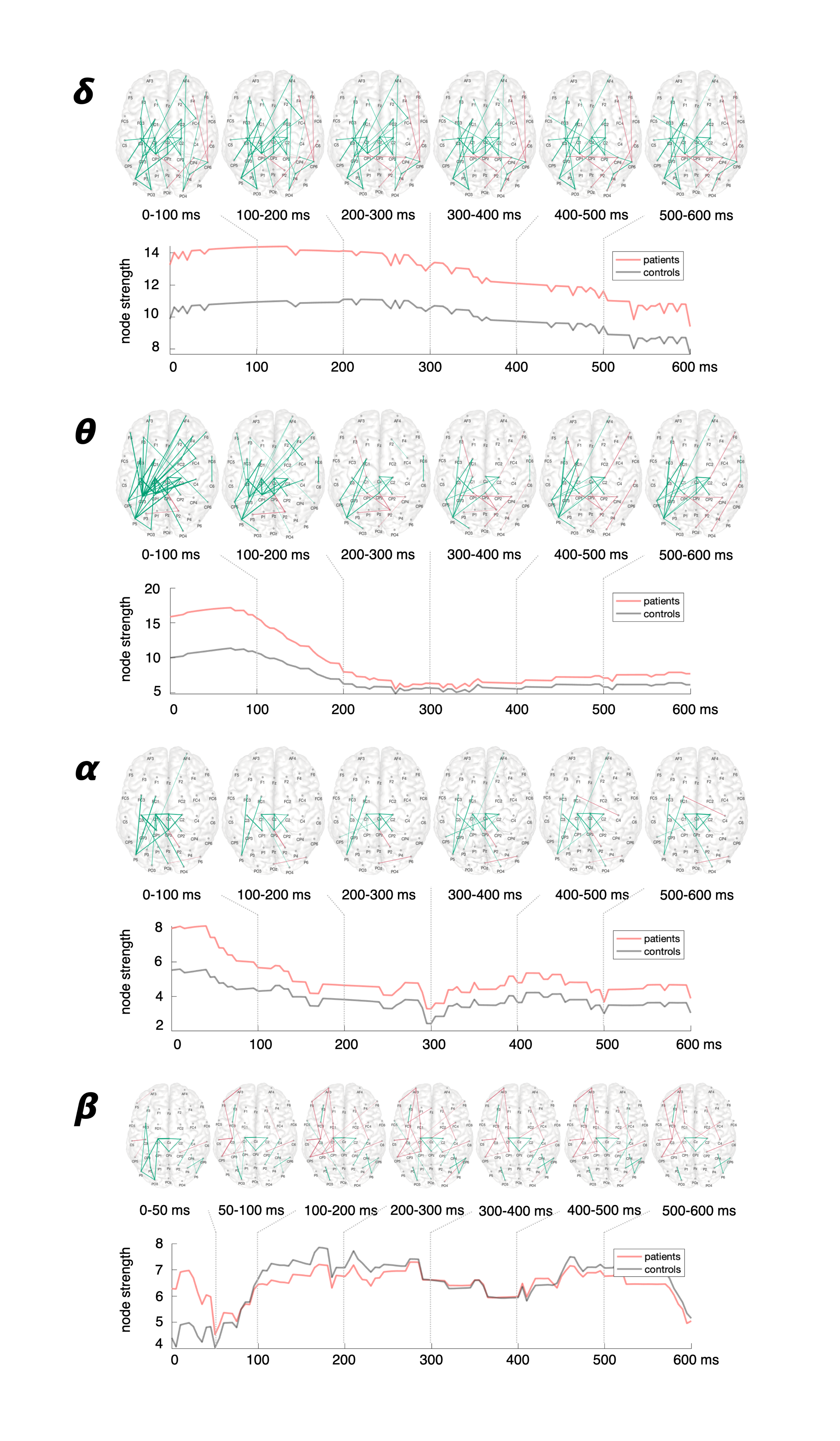
**

Dynamical analyses of the time course of node strength of the entire network connectivity suggested differential trajectories for each frequency after the TMS pulse. While within the first 50-100ms post-stimulus, stroke patients showed increased coupling and, thus, increased node strength in all frequency bands, this difference sustained throughout the time period after TMS onset predominantly for the delta frequency. Please note that for each frequency, the upper panel displays the dynamic network connections along the post-stimulus period (colors indicate between-group comparison of connectivity with green: stroke (n=41)>healthy participants (n=15) and magenta: stroke<healthy participants, p<0.05, FDR-corrected point-wise t-tests; left hemisphere=ipsilesional site, right hemisphere=contralesional site) and the lower panel depicts the time course of the overall node strength (red: patients, grey: control subjects).

**Supplementary Figure 3 – Increase of core nodes in the delta and theta frequency
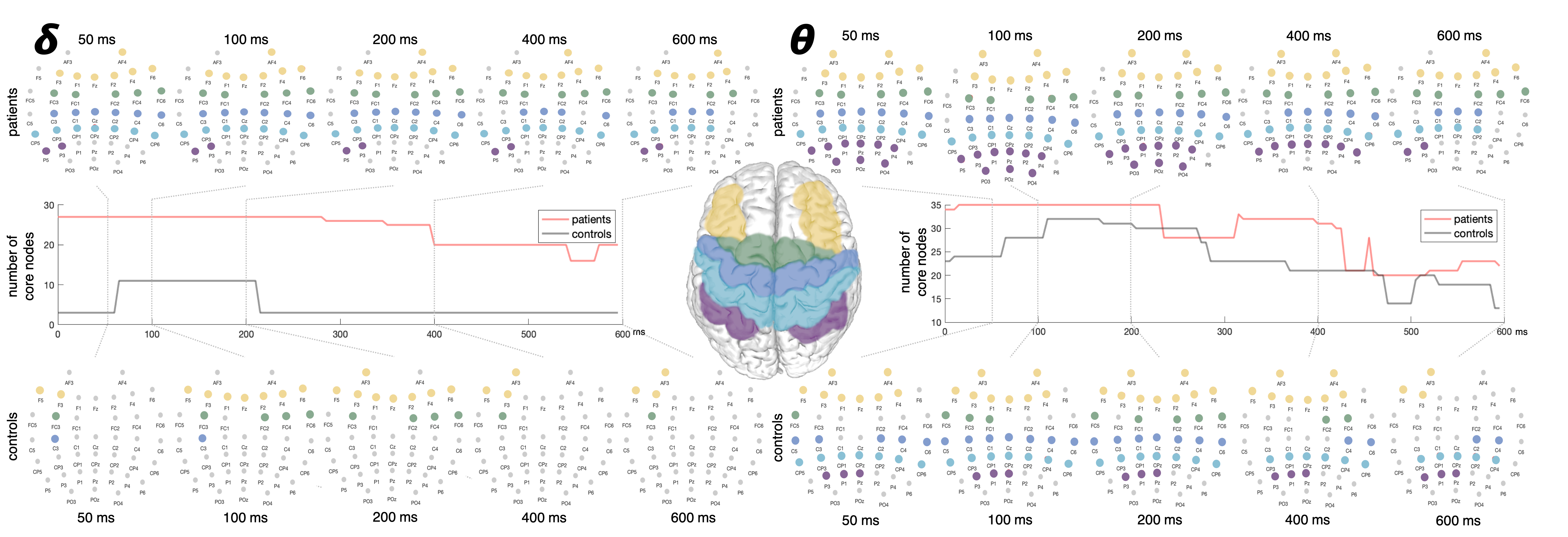
early post-stroke**

The significant core nodes over the time post-stimulus are illustrated with an extensive bihemispheric network topology that lasted up to 600 ms post-stimulus in the delta and theta-frequency in stroke patients. The upper panel displays the network core of stroke patients, in the middle the time course of significant core nodes for the entire post-stimulus period is depicted, and the lower panel shows the network of healthy controls. (between-group comparison t-test: stroke (n=41) and healthy participants (n=15); delta: p=0.011, t_(54.0)_=2.77; theta: p=0.011, t_(16.9)_=2.85; FDR-corrected; left hemisphere=ipsilesional site, right hemisphere=contralesional site)

| Patient No. | Lesion-side | Lesion location | Lesion volume | NIHSS | | | | | Rel. grip strength | | | | Motricity index | | | ARAT | | | | |
| --- | --- | --- | --- | --- | --- | --- | --- | --- | --- | --- | --- | --- | --- | --- | --- | --- | --- | --- | --- | --- |
|  |  |  |  | **Admission** | **Session I** | | **Session II** | | **Session I** | | **Session II** | | **Session I** | | **Session II** | | **Session I** | **Session II** | | |
| 1 | R | Striato-capsular | 4220 | 7 | | 9 | | 7 | | 0.0 | | 0.0 | | 19.5 | 25.5 | | 0 | | 0 |  |
| 2 | R | partial MCA | 10286 | 10 | | 7 | | 1 | | 0.0 | | 14.1 | | 58 | 86.0 | | 4 | | 50 |  |
| 3 | R | Lenticulo-striate, parieto-occipital | 5239 | 9 | | 10 | | 9 | | 0.0 | | 0.0 | | 13.5 | 13.5 | | 0 | | 0 |  |
| 4 | R | Lenticulo-striate | 3138 | 11 | | 8 | | 2 | | 19.3 | | 47.6 | | 49 | 79.5 | | 3 | | 57 |  |
| 5 | L | pons | 4129 | 15 | | 12 | |  | | 0.0 | |  | | 0 |  | | 0 | |  |  |
| 6 | R | partial MCA | 6418 | 4 | | 5 | | 3 | | 48.3 | | 59.9 | | 47 | 69.5 | | 27 | | 51 |  |
| 7 | L | basal ganglia | 4258 | 7 | | 7 | | 3 | | 2.3 | | 13.9 | | 16.5 | 67.5 | | 3 | | 7 |  |
| 8 | L | internal capsule | 864 | 2 | | 1 | | 1 | | 58.3 | | 38.8 | | 86.5 | 87.5 | | 54 | | 53 |  |
| 9 | L | partial MCA | 52540 | 3 | | 12 | | 6 | | 0.0 | | 0.0 | | 0 | 31.0 | | 0 | | 0 |  |
| 10 | R | Lenticulo-striate | 20201 | 1 | | 1 | | 0 | | 88.8 | | 70.4 | | 87.5 | 87.5 | | 54 | | 57 |  |
| 11 | R | MCA | 45966 | 11 | | 2 | | 0 | | 85.9 | | 100.0 | | 91.5 | 99.0 | | 55 | | 57 |  |
| 12 | R | pons | 519 | 6 | | 4 | | 0 | | 94.7 | | 91.3 | | 80 | 95.0 | | 48 | | 57 |  |
| 13 | R | partial MCA | 11470 | 7 | | 4 | | 0 | | 79.5 | | 86.2 | | 84.5 | 85.5 | | 45 | | 51 |  |
| 14 | L | centrum semiovale | 1839 | 1 | | 1 | |  | | 70.4 | |  | | 92 |  | | 53 | |  |  |
| 15 | R | partial MCA | 16614 | 5 | | 4 | | 0 | | 48.7 | | 92.2 | | 88.5 | 99.0 | | 48 | | 57 |  |
| 16 | L | basal ganglia | 771 | 7 | | 5 | | 2 | | 23.8 | | 77.9 | | 83 | 83.5 | | 47 | | 55 |  |
| 17 | L | pons | 4273 | 8 | | 12 | | 7 | | 0.0 | | 0.0 | | 0 | 45.5 | | 0 | | 0 |  |
| 18 | L | corona radiata | 2185 | 3 | | 10 | | 2 | | 0.0 | | 63.6 | | 18.5 | 81.5 | | 0 | | 20 |  |
| 19 | R | corona radiata | 2837 | 3 | | 10 | | 6 | | 0.0 | | 0.0 | | 16 | 52.0 | | 0 | | 3 |  |
| 20 | R | partial MCA | 95911 | 4 | | 2 | | 2 | | 80.0 | | 96.0 | | 90 | 95.5 | | 53 | | 54 |  |
| 21 | L | internal capsule | 926 | 1 | | 5 | | 1 | | 83.3 | | 58.3 | | 76.5 | 81.5 | | 31 | | 54 |  |
| 22 | R | basal ganglia | 71408 | 14 | | 11 | | 10 | | 0.0 | | 0.0 | | 41 | 29.0 | | 0 | | 0 |  |
| 23 | L | partial MCA | 187136 | 18 | | 14 | | 9 | | 0.0 | | 17.3 | | 14 | 48.0 | | 0 | | 4 |  |
| 24 | R | partial MCA | 21475 | 8 | | 4 | | 2 | | 67.2 | | 74.8 | | 77 | 99.0 | | 52 | | 57 |  |
| 25 | L | partial MCA | 9162 | 14 | | 8 | | 2 | | 65.8 | | 93.8 | | 61.5 | 99.0 | | 22 | | 56 |  |
| 26 | L | internal capsule | 1245 | 11 | | 7 | | 5 | | 29.3 | | 26.9 | | 45.5 | 67.5 | | 3 | | 38 |  |
| 27 | R | medulla oblongata | 324 | 7 | | 7 | |  | | 0.0 | |  | | 21.5 |  | | 3 | |  |  |
| 28 | L | Lenticulo-striate | 17384 | 36 | | 11 | | 9 | | 0.0 | | 0.0 | | 13.5 | 16.0 | | 0 | | 0 |  |
| 29 | L | partial MCA | 9831 | 6 | | 5 | | 2 | | 89.5 | | 101.4 | | 95.5 | 95.5 | | 47 | | 51 |  |
| 30 | R | partial MCA | 49616 | 15 | | 10 | | 2 | | 0.0 | | 11.1 | | 14 | 60.5 | | 0 | | 24 |  |
| 31 | R | thalamus | 1598 | 19 | | 7 | | 3 | | 78.8 | | 78.8 | | 67.5 |  | | 44 | | 55 |  |
| 32 | L | MCA | 293623 | 12 | | 10 | | 10 | | 0.0 | | 0.0 | | 4.5 | 9.0 | | 0 | | 0 |  |
| 33 | R | MCA | 32158 | 12 | | 11 | | 6 | | 0.0 | | 16.9 | | 11.5 | 52.5 | | 0 | | 31 |  |
| 34 | R | partial MCA | 4613 | 8 | | 3 | |  | | 43.5 | |  | | 79.5 |  | | 39 | |  |  |
| 35 | R | partial MCA | 81994 | 16 | | 9 | | 4 | | 0.0 | | 0.0 | | 28 | 44.5 | | 0 | | 3 |  |
| 36 | R | partial MCA, internal capsule | 24692 | 11 | | 9 | | 3 | | 0.0 | | 75.4 | | 13.5 | 83.5 | | 3 | | 57 |  |
| 37 | L | corona radiata | 2474 | 13 | | 12 | |  | | 0.0 | |  | | 4.5 |  | | 0 | |  |  |
| 38 | L | corona radiata | 5792 | 12 | | 11 | | 5 | | 0.0 | | 0.0 | | 4.5 | 54.0 | | 0 | | 13 |  |
| 39 | L | Lenticulo-striate | 2160 | 5 | | 8 | | 4 | | 0.0 | | 20.5 | | 30.5 | 79.5 | | 3 | | 35 |  |
| 40 | L | Lenticulo-striate | 1930 | 2 | | 8 | | 6 | | 0.0 | | 0.0 | | 4.5 |  | | 0 | | 0 |  |
| 41 | R | corona radiata | 1010 | 10 | | 10 | | 6 | | 0.0 | | 0.0 | | 11.5 |  | | 0 | | 0 |  |
| MEAN  SD |  |  | **27176.32**  **55466.67** | **9.1**  **6.4** | | **7.5**  **3.5** | | **3.8**  **3.1** | | **28.3**  **35.7** | | **39.6**  **38.4** | | **42.5**  **34.0** | **66.8**  **27.7** | | **18.0**  **22.7** | | **32.6**  **24.4** |  |

**Supplementary Table 1 – Patient characteristics**

**REFERENCES**

1. Nikouline V, Ruohonen J, Ilmoniemi RJ. The role of the coil click in TMS assessed with simultaneous EEG. *Clin Neurophysiol*. 1999;110(8):1325-1328. doi:10.1016/s1388-2457(99)00070-x

2. Ilmoniemi RJ, Kičić D. Methodology for Combined TMS and EEG. *Brain Topogr*. 2009;22(4):233. doi:10.1007/s10548-009-0123-4

3. Braack EM ter, Vos CC de, Putten MJAM van. Masking the Auditory Evoked Potential in TMS-EEG: A Comparison of Various Methods. *Brain Topogr*. 2015;28(3):520-528. doi:10.1007/s10548-013-0312-z

4. Belardinelli P, Biabani M, Blumberger DM, et al. Reproducibility in TMS-EEG studies: A call for data sharing, standard procedures and effective experimental control. *Brain Stimul*. 2019;12(3):787-790. doi:10.1016/j.brs.2019.01.010

5. Tscherpel C, Dern S, Hensel L, Ziemann U, Fink GR, Grefkes C. Brain responsivity provides an individual readout for motor recovery after stroke. *Brain*. 2020;143(6):awaa127-. doi:10.1093/brain/awaa127

6. Massimini M, Ferrarelli F, Huber R, Esser SK, Singh H, Tononi G. Breakdown of cortical effective connectivity during sleep. *Science*. 2005;309(5744):2228-2232. doi:10.1126/science.1117256

7. Massimini M, Ferrarelli F, Esser SK, et al. Triggering sleep slow waves by transcranial magnetic stimulation. *Proc National Acad Sci*. 2007;104(20):8496-8501. doi:10.1073/pnas.0702495104

8. Fecchio M, Pigorini A, Comanducci A, et al. The spectral features of EEG responses to transcranial magnetic stimulation of the primary motor cortex depend on the amplitude of the motor evoked potentials. *PLoS ONE*. 2017;12(9):e0184910. doi:10.1371/journal.pone.0184910.s005

9. Casali AG, Casarotto S, Rosanova M, Mariotti M, Massimini M. General indices to characterize the electrical response of the cerebral cortex to TMS. *Neuroimage*. 2010;49(2):1459-1468. doi:10.1016/j.neuroimage.2009.09.026

10. Rosanova M, Casali A, Bellina V, Resta F, Mariotti M, Massimini M. Natural Frequencies of Human Corticothalamic Circuits. *J Neurosci*. 2009;29(24):7679-7685. doi:10.1523/jneurosci.0445-09.2009

11. Komssi S, Kähkönen S, Ilmoniemi RJ. The effect of stimulus intensity on brain responses evoked by transcranial magnetic stimulation. *Hum Brain Mapp*. 2004;21(3):154-164. doi:10.1002/hbm.10159

12. Rosanova M, Fecchio M, Casarotto S, et al. Sleep-like cortical OFF-periods disrupt causality and complexity in the brain of unresponsive wakefulness syndrome patients. *Nat Commun*. 2018;9(1):1-10. doi:10.1038/s41467-018-06871-1

13. Casarotto S, Comanducci A, Rosanova M, et al. Stratification of unresponsive patients by an independently validated index of brain complexity. *Brain Injury*. 2016;80(5):718-729. doi:10.3109/02699052.2012.698362

14. Rogasch NC, Sullivan C, Thomson RH, et al. Analysing concurrent transcranial magnetic stimulation and electroencephalographic data: A review and introduction to the open-source TESA software. *Neuroimage*. 2016;147:934-951. doi:10.1016/j.neuroimage.2016.10.031

15. Delorme A, Makeig S. EEGLAB: an open source toolbox for analysis of single-trial EEG dynamics including independent component analysis. *J Neurosci Meth*. 2004;134(1):9-21. doi:10.1016/j.jneumeth.2003.10.009

16. Rogasch NC, Thomson RH, Farzan F, et al. Removing artefacts from TMS-EEG recordings using independent component analysis: Importance for assessing prefrontal and motor cortex network properties. *NeuroImage*. 2014;101(C):425-439. doi:10.1016/j.neuroimage.2014.07.037

17. Kayser J, Tenke CE. Principal components analysis of Laplacian waveforms as a generic method for identifying ERP generator patterns: I. Evaluation with auditory oddball tasks. *Clin Neurophysiol*. 2006;117(2):348-368. doi:10.1016/j.clinph.2005.08.034

18. Hjorth B. An on-line transformation of EEG scalp potentials into orthogonal source derivations. *Electroencephalogr Clin Neurophysiol*. 1975;39(5):526-530. doi:10.1016/0013-4694(75)90056-5

19. Lachaux J, Rodriguez E, Martinerie J, Varela FJ. Measuring phase synchrony in brain signals. *Hum Brain Mapp*. 1999;8(4):194-208. doi:10.1002/(sici)1097-0193(1999)8:4<194::aid-hbm4>3.0.co;2-c

20. Rosjat N, Liu L, Wang BA, et al. Aging-associated changes of movement-related functional connectivity in the human brain. *Neuropsychologia*. 2018;117:520-529. doi:10.1016/j.neuropsychologia.2018.07.006

21. Rosjat N, Wang BA, Liu L, Fink GR, Daun S. Stimulus transformation into motor action: Dynamic graph analysis reveals a posterior‐to‐anterior shift in brain network communication of older subjects. *Hum Brain Mapp*. 2021;42(5):1547-1563. doi:10.1002/hbm.25313
